# Effectiveness and safety of tenofovir alafenamide in chronic hepatitis B patients over 30 years old with positive hepatitis B virus DNA: a double-center retrospective study

**DOI:** 10.3389/fmed.2025.1680839

**Published:** 2025-11-12

**Authors:** Yinong Feng, Li Zhou, Shaoyuan Shi, Zehong Wang, Xuanxuan Wang, Fan Du

**Affiliations:** 1 Department of Hepatology, The Third People’s Hospital of Taiyuan, Taiyuan, China; 2 Department of Gastroenterology, Union Hospital, Tongji Medical College, Huazhong University of Science and Technology, Wuhan, China

**Keywords:** chronic hepatitis B, tenofovir alafenamide, effectiveness, HBV DNA, retrospective study

## Abstract

**Aims:**

The aim of this study was to evaluate the effectiveness and safety of Tenofovir Alafenamide (TAF) in chronic hepatitis B (CHB) patients, particularly those aged 30–35 years or with baseline normal alanine aminotransferase (ALT) levels.

**Methods:**

A total of 191 patients were recruited, and their data were collected from two hospital in China from January 2017 to March 2023. Liver function and HBV-related indicators were measured at baseline, 48-week, and 96-week. The safety and effectiveness of TAF were evaluated in the high-age group (> 35 years) and the low-age group (30–35 years), as well as in the ALT-normal group (ALT < 1 × ULN) and the ALT-elevated (ALT ≥ 1 × ULN) group.

**Results:**

TAF treatment for 48 weeks or 96 weeks could significantly improve the progression of hepatitis as evidenced by lower ALT, AST, and HBV DNA. TAF is still effective for patients aged 30–35 or those with normal ALT levels. Additionally, for CHB patients with baseline chronic kidney disease staging at stage 2 or below, 1–2 years of TAF treatment has minimal impact on their renal function.

**Conclusion:**

TAF treatment significantly alleviated the progression of CHB patients over a 96-week follow-up period. TAF remains effective for younger patients or those with normal liver function, providing evidence for further expanding antiviral indications.

## 1 Introduction

Chronic hepatitis B (CHB) infection remains a major and pressing global public health problem. It is estimated that approximately 316 million people were suffering from CHB infection in 2019, globally, and about 555 thousand people succumbed to the diseases related with hepatitis B virus (HBV) infection such as cirrhosis, liver failure and hepatocellular carcinoma (HCC) (1). The essential treatment of CHB is antiviral therapy, which can maximally inhibit the replication of HBV, thereby lowering the incidence of end-stage liver disease and improving the life quality (2–4). However, antiviral treatment has historically been restricted to patients with elevated alanine aminotransferase (ALT), established cirrhosis, demonstration of significant liver necroinflammation or fibrosis and/or the risk of HCC development. As a consequence, many patients have been excluded from antiviral treatment, whereas some previous studies suggested that these untreated patients also have a risk of disease progression (5, 6). To achieve the World Health Organization (WHO) goal to tackle the burden of viral hepatitis by the year 2030, there has been a growing expansion of the screening and treatment for HBV infection in current international guidelines (7–9).

China has the largest population of CHB around the world, and there has been more and more patients suffering progressive liver histological changes during clinical follow-up, who were formerly considered to be in a benign disease phase and not require antiviral treatment. With the recent advances in understanding of CHB and the accessibility of potent and safe nucleos(t)ide analogs (NAs), the Chinese Society of Infectious Diseases has further scaled up the indications for antiviral therapy in the updated guidelines for the prevention and treatment of CHB (version 2022) (10). Firstly, guidance on treatment initiation of the ALT threshold was lowered to 30 U/L for male and 19 U/L for female, indicating that abnormal ALT is not a necessary factor for starting treatment. And for patients with positive serum HBV DNA, regardless of ALT levels, antiviral therapy is recommended as long as one of the following conditions is met: (a) family history of hepatitis B cirrhosis or HCC; (b) older age (over 30 years); (c) the demonstration of significant liver necroinflammation or fibrosis assessed by invasive or non-invasive examination; (d) HBV related extrahepatic manifestations. It is worth noting that there were different opinions in the previous guidelines of Chinese Society of Infectious Diseases (11). In addition to suggesting lowering the treatment threshold for ALT, experts also advocate that patients with positive serum HBV DNA and over 30 years old should receive antiviral therapy regardless of ALT levels.

With increasing researches indicating that age over 30 years is an independent risk factor for HBV-related disease progression (5, 12), age also plays a critical role in determining antiviral treatment initiation in patients with positive serum HBV DNA. Considering there are currently not so many clinical studies to explore the necessity of antiviral therapy for CHB patients over 30 years old with positive serum HBV DNA, our study aims to evaluate the effectiveness and safety of antiviral treatment (taking TAF) among these specific population.

## 2 Materials and methods

### 2.1 Study population and study design

The CHB patients who were attended at the Department of Hepatology of the Third People’s Hospital of Taiyuan and the Department of Gastroenterology of the Union Hospital of Tongji Medical College of Huazhong University of Science and Technology during January 2017 and March 2023 were enrolled in this study. Inclusion criteria: (1) age ≥ 30 years; (2) meeting the CHB diagnostic criteria of the guidelines: a history of hepatitis B or detectable hepatitis B surface antigen (HBsAg) for at least 24 weeks, and currently detectable HBsAg and/or HBV DNA (upper limit of normal of HBV DNA of 20 IU/mL); and (3) without previous NAs or interferon antiviral treatment. Exclusion criteria: (1) presence of other chronic liver diseases, such as alcoholic liver disease, drug-induced liver disease or autoimmune liver disease, etc., or liver cirrhosis and HCC; (2) co-infection with hepatitis A virus, hepatitis C virus, hepatitis D virus, hepatitis E virus or human immunodeficiency virus; (3) pregnant; or (4) follow-up period less than 48 weeks. All study populations received TAF to initiate antiviral therapy and were categorized into two subgroups based on their age or ALT levels at the baseline. Eventually, a total of 191 patients were included in the baseline and 48-week data analysis; 64 patients were lost to follow-up by week 96, resulting in a total of 127 patients followed up.

### 2.2 Data collection

The clinical data were collected at 48 and 96 weeks after initial antiviral therapy. Including age and gender as demographic characteristics. Serum laboratory parameters included serum HBV DNA concentration with a lower limit of detection of 20 IU/mL, serum HBsAg status (> 0.05 IU/mL was positive) and serum hepatitis B e antigen (HBeAg) status (> 1 S/CO was positive), ALT level, aspartate aminotransferase (AST) level, total bilirubin (TBIL) level, platelet (PLT) count, total cholesterol (TCh) level, creatinine (Cr) level and estimated glomerular filtration rate (eGFR). All serum laboratory indicators were completed in the testing center of the Department of Hepatology of the Third People’s Hospital of Taiyuan and the Department of Gastroenterology of the Union Hospital of Tongji Medical College of Huazhong University of Science and Technology.

### 2.3 Outcomes

The primary outcome was complete virological response (CVR), which was defined as serum HBV DNA < 20 IU/mL after antiviral treatment. Secondary assessments included the following: (1) HBsAg negative conversion rate, HBeAg negative conversion rate and the recovery rate of ALT after antiviral treatment. The HBsAg Reagent Kit was purchased from Abbott Ireland Diagnostics Division (the detection limit is 0.05 IU/mL) and used for diagnosis according to the instructions. (2) changes of chronic kidney disease (CKD) stages graded by eGFR levels: serum eGFR levels ≥ 90 mL/min/1.73 m^2^ as Phase I, serum eGFR levels range from 60 to 89 mL/min/1.73 m^2^ as Phase II; serum eGFR levels range from 30 to 59 mL/min/1.73 m^2^ as Phase III, serum eGFR levels range from 15 to 29 mL/min/1.73 m^2^ as Phase IV, and serum eGFR levels < 15 mL/min/1.73 m^2^ as Phase V.

### 2.4 Sample size calculation

Previous studies showed that the CVR rate to TAF treatment in CHB patients ranged from 60 to 80%. This study adopted a median value of 70% for sample size estimation based on α = 0.05, β = 0.2, and a dropout rate of 20%, yielding a required sample size of 114. Ultimately, 127 patients were followed up in this study, meeting the statistical requirements.

### 2.5 Statistical analysis

SPSS version 22 statistical software was used for data analysis. Categorical variables were expressed as frequencies (percentages) and continuous variables were reported as mean ± standard deviation or median with interquartile range (IQR). Comparison between frequencies and distributions of categorical variables was performed using chi square test or and Fisher’s exact test. Continuous variables were compared with Student’s *t*-test or Mann-Whitney U test. *P* < 0.05 was considered statistically significant. If the missing value of a continuous variable was less than 10%, the missing value was filled by multiple mean imputation.

## 3 Results

### 3.1 Baseline clinical characteristics of CHB patients overall and in subgroups

**FIGURE 1:**
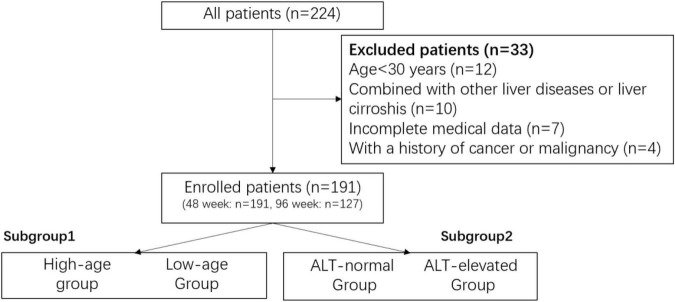
The screen chart.

After screening (Figure 1), a total of 191 patients with CHB were included in this study, and their mean age was 45.05 years old, with a higher proportion of males (65.4%). A majority of patients were older than 35 years old (81.2%) and a predominantly stage I CKD classification (90.1%). Regarding hepatitis B-related indicators, the percentage of patients with baseline ALT < 40 U/L was 59.2%, 30.4% had HBV DNA < 2,000 IU/mL, 24.1% were in the range of 2,000 to 20,000 IU/mL, while 45.5% had more than 20,000 IU/mL, and all the patients were HBsAg-positive, and 63.9% were HBeAg-negative patients. The rest of the laboratory tests are shown in Table 1.

**TABLE 1:** The baseline data of the enrolled CHB patients.

Items	All patients (*n* = 191)	Subgroup 1 low-age group (*n* = 36)	High-age group (*n* = 155)	*P*-value	Subgroup2 ALT-normal group (*n* = 113)	ALT-elevated group (*n* = 78)	*P*-value
Gender (male, %)	125, 65.4%	23,63.9%	102, 65.8%	0.847	66, 58.4%	59, 75.6%	**0.020**
Age	45.05 ± 9.39	33.17 ± 1.63	47.81 ± 8.22	/	47.03 ± 9.44	42.18 ± 8.59	**< 0.001**
30–35	36, 18.8%	/	/	/	15, 13.3%	21, 26.9%	**0.024**
> 35	155, 81.2%	/	/		98, 86.7%	57, 73.1%	
PLT(G/L)	184.95 ± 59.70	194.23 ± 52.53	182.77 ± 61.23	0.315	192.12 ± 52.40	175.24 ± 67.52	0.061
TB (μmol/L)	19.04 ± 24.08	17.17 ± 6.20	19.48 ± 26.57	0.606	15.97 ± 7.52	23.50 ± 36.26	0.074
ALT (U/L)	51.79 ± 65.26	57.62 ± 42.07	50.43 ± 69.59	0.553	23.95 ± 8.52	92.12 ± 87.29	/
≤ 40	113, 59.2%	15, 41.7%	98, 63.2%	**0.024**	/	/	/
>40	78, 40.8%	21,58.3%	57, 36.8%		/	/	
AST (U/L)	42.44 ± 63.15	40.08 ± 27.23	42.99 ± 68.92	0.804	23.27 ± 6.71	70.22 ± 91.95	**< 0.001**
TC (mmol/L)	4.71 ± 1.80	4.41 ± 0.89	4.78 ± 1.94	0.331	4.66 ± 0.86	5.01 ± 4.78	0.676
TG (mmol/L)	1.31 ± 0.97	1.12 ± 1.07	1.36 ± 0.94	0.234	1.24 ± 0.96	1.41 ± 0.99	0.276
CR (μmol/L)	68.10 ± 15.28	64.37 ± 13.32	68.92 ± 15.60	0.134	69.06 ± 17.59	66.82 ± 11.54	0.344
eGFR mL/(min/1.73 m^2^)	114.78 ± 26.52	121.65 ± 35.98	113.18 ± 23.66	0.084	106.95 ± 18.85	126.12 ± 31.60	**< 0.001**
**HBV DNA (IU/mL)**							
≤ 2,000	58, 30.4%	9, 25.0%	49, 31.6%	**0.033**	46, 40.7%	12, 15.4%	**< 0.001**
2,000–20,000	46, 24.1%	4, 11.1%	42, 27.1%		30, 26.5%	16, 29.5%	
>20,000	87, 45.5%	23, 63.9%	64, 41.3%		37, 32.7%	50, 64.1%	
Lg HBV DNA (IU/mL)	4.0170 (3.1931–5.9420)	7.1474 (3.2253–8.0000)	3.8831 (3.1931–5.5866)	**0.003**	3.5999 (2.9585,5.3284)	5.5371 (3.5911,7.5855)	**< 0.001**
Positive HBsAg	191, 100%	36, 100%	155, 100%	/	113, 100%	78, 100%	/
Positive HBeAg	69, 36.1%	22, 61.1%	47, 30.3%	**0.001**	26, 23/0%	43, 55.1%	**< 0.001**
**CKD level**							
Phase I	172, 90.1%	34, 94.4%	138, 89.0%	0.597	97, 85.8%	75, 96.2%	0.061
Phase II	18, 9.4%	2, 5.6%	16, 10.3%		15, 13.3%	3, 3.8%	
Phase III	1, 0.5%	0, 0%	1, 0.6%		1, 0.9%	1, 0.5%	

Bold numerals indicate differences that were statistically significant.

Subsequently, this study firstly analyzed the included patients in subgroups according to their age, in which CHB patients with age less than 35 years old were considered as the low-age group (*n* = 36), and those with age greater than 35 years old were the high-age group (*n* = 155). At baseline, the level of HBV DNA, HBeAg positivity rate and abnormal ALT rate were significantly higher in the low-age group than in the high-age group, and the differences in the rest of the indicators were not statistically significant (Table 1), indicating that with the age of CHB patients, their hepatitis B virus level and e antigen level showed a gradual trend of decreasing, which was in line with the natural history of CHB, i.e., HBV DNA levels gradually decrease and HBeAg may seroconvert to negative with increasing age.

The subgroups were also analyzed based on the ALT levels at the time of patient enrollment, where those with baseline ALT ≤ 40 U/L were considered the ALT-normal group (*n* = 118) and those with > 40 U/L were considered the ALT-elevated group (*n* = 78). Compared with the ALT-elevated group, the ALT-normal group was older, had lower AST, eGFR, HBV DNA, and smaller proportion of males, and the rest of the indices did not show significant differences (Table 1). All of the above patients were treated regularly with TAF for 48–96 weeks, and the specific effectiveness of the treatment is described later.

### 3.2 Effectiveness of TAF in CHB patients after 48-week treatment

#### 3.2.1 The 48-week effectiveness in all enrolled CHB patients

Compared with the baseline, the level of AST, the ALT abnormality rate and the HBV DNA positive rate of CHB patients decreased significantly after 48-week TAF treatment, 70.7% of HBV DNA was undetectable, and no patient showed negative conversion of HBsAg, but there was a trend of decreasing HBeAg positive rate. In addition, there was no significant difference for the indices of blood lipid and CKD grading (Table 2). This result suggested that TAF treatment for 48 weeks could significantly improve the progression of hepatitis and had no adverse effects on lipid metabolism. For CHB patients with baseline chronic kidney disease staging at stage 2 or below, 1 year of TAF treatment has minimal impact on their renal function.

**TABLE 2:** The 48-week effectiveness and safety of TAF in all CHB patients.

	All patients (*n* = 191)		
Items	Baseline	48-week	*P*-value
**ALT (U/L)**			
≤ 40	113, 59.2%	116, 86.9%	**<0.001**
40–80	78, 40.8%	25, 13.1%	**<0.001**
Positive HBV DNA	191, 100%	56, 29.3%	**<0.001**
Positive HBsAg	191, 100%	191, 100%	/
Positive HBeAg	69, 36.1%	62, 32.5%	0.518
**CKD level**			
Phase I	172, 90.1%	169, 88.5%	0.536
Phase II	18, 9.4%	20, 10.5%	
Phase III	1, 0.5%	0, 0%	
Phase IV	0, 0%	1, 0.5%	
Phase V	0, 0%	1, 0.5%	
PLT (G/L)	184.95 ± 59.70	184.35 ± 56.75	0.923
AST (U/L)	42.44 ± 63.15	26.19 ± 16.31	**<0.001**
TC (mmol/L)	4.71 ± 1.80	4.68 ± 1.26	0.868
TG (mmol/L)	1.31 ± 0.97	1.33 ± 1.05	0.901

Bold numerals indicate differences that were statistically significant.

#### 3.2.2 Comparison of 48-week effectiveness between the high-age and low-age groups

We further focused on the effect of age on the outcome of TAF treatment. Totally, 155 patients in the high-age group and 36 in the low-age group were followed up for 48 weeks (Table 3). Compared with the baseline, ALT improved significantly in both two groups, with 88.4% of ALT normalized in the high-age group and 80.6% in the low-age group. The level of AST and HBV DNA positive rate decreased significantly as well. The HBsAg positive rate in both groups was unchanged and remained at 100%, but there was a decreasing trend in the HBeAg positive rate in both groups. In addition, PLT, TC, TG, and CKD grading of patients in both groups did not show significant changes before and after treatment, indicating that TAF is still effective for low-age CHB patients.

**TABLE 3:** The 48-week effectiveness and safety of TAF in the low-age group and the high-age group.

	Low-age group (*n* = 36)			High-age group (*n* = 155)		
Items	Baseline	48-week	*P*-value	Baseline	48-week	*P*-value
**ALT (U/L)**						
≤ 40	15, 41.7%	29, 80.6%	**0.001**	98, 63.2%	137, 88.4%	**< 0.001**
40–80	12, 33.3%	7, 19.4%		27, 17.4%	12, 7.7%	
> 80	9, 25.0%	0, 0%		30, 19.4%	6, 3.9%	
Positive HBV DNA	36, 100%	21, 58.3%	**< 0.001**	155, 100%	35, 22.6%	**< 0.001**
Positive HBsAg	36, 100%	36, 100%	/	155, 100%	155, 100%	/
Positive HBeAg	22, 61.1%	20, 55.6%	0.811	47, 30.3%	42, 27.1%	0.616
**CKD level**						
Phase I	34, 94.4%	35, 97.2%	0.555	138, 89.0%	134, 86.5%	0.506
Phase II	2, 5.6%	1, 2.8%		16, 10.3%	19, 12.3%	
Phase III	0, 0%	0, 0%		1, 0.6%	0, 0.0%	
Phase IV	0, 0%	0, 0%		0, 0%	1, 0.6%	
Phase V	0, 0%	0, 0%		0, 0%	1, 0.6%	
PLT (G/L)	194.23 ± 52.53	185.13 ± 50.43	0.479	182.77 ± 61.23	184.18 ± 58.20	0.842
AST (U/L)	40.08 ± 27.23	26.17 ± 9.78	**0.006**	42.99 ± 68.92	26.19 ± 17.51	**0.004**
TC (mmol/L)	4.41 ± 0.89	4.44 ± 0.97	0.909	4.78 ± 1.94	4.73 ± 1.31	0.816
TG (mmol/L)	1.12 ± 1.07	1.15 ± 1.23	0.921	1.36 ± 0.94	1.36 ± 1.01	0.959

Bold numerals indicate differences that were statistically significant.

#### 3.2.3 Comparison of 48-week effectiveness between the ALT-normal and ALT-elevated groups

We also evaluated the effect of baseline ALT on the outcome of TAF treatment. There were 113 patients in the normal-ALT group and 78 in the elevated-ALT group, respectively (Table 4). Compared with baseline, both groups showed a significant decrease in HBV DNA positive rate, and there was no change in HBsAg positive rate but a decreasing trend in HBeAg positive rate. There were no significant differences in PLT, AST, TC and TG before and after treatment. The above results indicated that TAF remained effective for CHB patients whose baseline ALT levels are normal.

**TABLE 4:** The 48-week effectiveness and safety of TAF in the ALT-normal group and the ALT-elevated group.

	ALT-normal group (*n* = 113)			ALT-elevated group (*n* = 78)		
Items	Baseline	48-week	*P*-value	Baseline	48-week	*P*-value
Positive HBV DNA	113, 100%	25, 22.1%	**< 0.001**	78,100%	31, 39.7%	**< 0.001**
Positive HBsAg	113, 100%	113, 100%	/	78, 100%	78, 100%	/
Positive HBeAg	26, 23.0%	24, 21.2%	0.873	43, 55.1%	38, 48.7%	0.522
PLT (G/L)	192.12 ± 52.40	189.39 ± 50.37	0.705	175.24 ± 67.52	177.29 ± 64.36	0.850
TC (mmol/L)	4.66 ± 0.86	4.69 ± 1.38	0.853	4.78 ± 2.55	4.67 ± 1.10	0.748
TG (mmol/L)	1.24 ± 0.96	1.25 ± 1.08	0.913	1.41 ± 0.99	1.42 ± 1.02	0.927

Bold numerals indicate differences that were statistically significant.

### 3.3 Effectiveness of TAF in CHB patients after 96-week treatment

#### 3.3.1 The 96-week effectiveness in all enrolled CHB patients

Compared with baseline levels, the AST level, the ALT abnormal rate and the HBV DNA positive rate were significantly reduced in CHB patients after 96-week of TAF treatment, while no significant abnormalities were seen in lipids and CKD grading (Table 5). This result was similar at 48 weeks of treatment, suggesting that 96-week of TAF treatment could improve the disease progression of CHB patients in general. Moreover, TAF did not affect the lipid metabolism, and for CHB patients with CKD at stage 2 or lower at baseline, 2 years of TAF treatment showed minimal impact on renal function. Next, we further discussed the effects of age and baseline ALT level on the treatment outcome, respectively.

**TABLE 5:** The 96-week effectiveness and safety of TAF in all CHB patients.

	All patients (*n* = 127)		
Items	Baseline	96-week	*P*-value
**ALT (U/L)**			
≤ 40	73, 57.5%	109, 85.8%	**<0.001**
>40	54, 42.5%	18, 14.2%	**<0.001**
Positive HBV DNA	127, 100%	23, 18.1%	**<0.001**
Positive HBsAg	127, 100%	123, 96.9%	**0.018**
Positive HBeAg	53, 41.7%	44, 34.6%	0.358
**CKD level**			
Phase I	112, 88.2%	112, 88.2%	/
Phase II	15, 11.8%	15, 11.8%	
PLT (G/L)	184.94 ± 63.19	187.15 ± 63.91	0.907
AST (U/L)	47.38 ± 75.45	23.70 ± 10.20	**0.001**
TC (mmol/L)	4.65 ± 0.97	4.62 ± 1.00	0.951
TG (mmol/L)	1.27 ± 0.97	1.52 ± 1.99	0.397

Bold numerals indicate differences that were statistically significant.

#### 3.3.2 Comparison of 96-week effectiveness between the high-age and low-age groups

There were 109 patients in the high-age group and 18 in the low-age group followed up after 96-week of TAF treatment (Table 6). Compared with baseline, both the high- and low-age groups showed a significant increase in ALT normalization rate to more than 80%, and a significant decrease in AST and HBV DNA positive rates, but the difference in the amplitude of AST decrease between the two groups was not statistically significant. There was a decreasing trend of HBsAg and HBeAg positive rates in the low-age group, but the HBsAg positive rate in the high-age group decreased significantly. In addition, PLT, TC, TG and CKD grading of both groups did not show significant changes before and after treatment. It suggested that the age of CHB patients at baseline did not affect the effectiveness of TAF, which was still effective for CHB patients aged 30–35.

**TABLE 6:** The 96-week effectiveness and safety of TAF in the low-age group and the high-age group.

	Low-age group (*n* = 18)			High-age group (*n* = 109)		
Items	Baseline	96-week	*P*-value	Baseline	96-week	*P*-value
**ALT (U/L)**						
≤ 40	6, 33.3%	15, 83.3%	**0.001**	67, 61.5%	94, 86.2%	**< 0.001**
>40	12, 66.7%	3, 16.7%		42, 38.5%	15, 13.8%	
Positive HBV DNA	18, 100%	7, 38.9%	**< 0.001**	109, 100%	16, 14.7%	**< 0.001**
Positive HBsAg	18, 100%	17, 94.4%	0.361	109, 100%	106, 97.2%	**0.048**
Positive HBeAg	12, 66.7%	11, 61.1%	0.585	41, 37.6%	33, 30.3%	0.455
**CKD level**						
Phase I	18, 100%	18, 100%	/	94, 86.2%	94, 86.2%	/
Phase II	0, 0%	0, 0%		15, 13.8%	15, 13.8%	
PLT (G/L)	200.44 ± 64.80	182.13 ± 54.78	0.470	182.33 ± 62.85	187.92 ± 65.41	0.977
AST (U/L)	47.39 ± 33.13	21.96 ± 9.54	**0.001**	47.37 ± 80.42	23.99 ± 10.31	**0.001**
TC (mmol/L)	4.49 ± 1.09	4.31 ± 0.99	0.655	4.68 ± 0.95	4.67 ± 1.00	0.767
TG (mmol/L)	1.25 ± 1.34	1.10 ± 0.62	0.362	1.27 ± 0.90	1.59 ± 2.12	0.517

Bold numerals indicate differences that were statistically significant.

#### 3.3.3 Comparison of 96-week effectiveness between the ALT-normal and ALT-elevated groups

73 patients in the ALT-normal group and 54 patients in the ALT-elevated group were followed up after 96-week of TAF treatment (Table 7). Compared with the baseline indices, patients in the ALT-normal group showed a significant decrease in HBV DNA and HBsAg positive rates, and a downward trend in the rate of HBeAg positivity, whereas no significant differences were seen for PLT, AST, TC, and TG indices. Similarly, patients in the group with elevated ALT had a significant decrease in AST levels and HBV DNA positive rate, a decreasing trend in HBsAg and HBeAg positive rates, and no significant changes in PLT, TC, and TG levels. This indicated that TAF remained effective even for CHB patients with baseline normal ALT levels. The antiviral treatment did not adversely affect the renal function and blood lipids of CHB patients with different ALT levels.

**TABLE 7:** The 96-week effectiveness and safety of TAF in the ALT-normal group and the ALT-elevated group.

	ALT-normal group (*n* = 73)			ALT-elevated group (54)		
Items	Baseline	96-week	*P*-value	Baseline	96-week	*P*-value
Positive HBV DNA	73, 100%	13, 17.8%	**< 0.001**	54, 100%	10, 18.5%	**< 0.001**
Positive HBsAg	73, 100%	70, 95.9%	**0.048**	54, 100%	53, 98.1%	0.366
Positive HBeAg	21, 28.8%	18, 24.7%	0.732	32, 59.3%	26, 48.1%	0.411
PLT (G/L)	191.51 ± 55.44	186.66 ± 51.48	0.563	176.30 ± 71.76	187.75 ± 76.91	0.397
AST (U/L)	23.36 ± 7.61	23.90 ± 11.16	0.095	79.85 ± 107.63	23.44 ± 8.83	**< 0.001**
TC (mmol/L)	4.74 ± 0.90	4.57 ± 0.81	0.909	4.54 ± 1.05	4.68 ± 1.22	0.999
TG (mmol/L)	1.24 ± 1.01	1.57 ± 2.56	0.784	1.30 ± 0.93	1.46 ± 0.78	0.443

Bold numerals indicate differences that were statistically significant.

### 3.4 Hepatic inflammation and fibrosis in CHB patients with normal ALT

Recent studies showed that CHB patients with normal ALT and positive HBV DNA also had varying degrees of hepatic inflammation and fibrosis, so we performed liver biopsies on 37 patients. They were aged 36.27 ± 12.54 years, with ALT (36.89 ± 23.20) IU/mL and a male/female ratio of 1.31/1. The stage of liver fibrosis ranged from S0 to S4, with a predominance of S0–S1 in 20 cases (54%), and there were 17 patients with S ≥ 2 (46%), 10 cases with S2 (27%), S3 in 3 cases (8%), and S4 in 4 cases (11%). Liver inflammatory activity was graded between G0 and G4, with G0–G1 predominating in 29 cases (78%) and G ≥ 2 in 8 patients (22%). A liver puncture definitively diagnosed 31 cases of chronic hepatitis B and 6 cases of cirrhosis. This result suggested that antiviral therapy was necessary even for CHB patients with normal ALT.

## 4 Discussion

In this study, we found that TAF significantly alleviated the progression of hepatitis B in patients treated with TAF over a 96-week follow-up period. In addition, for CHB patients aged 30–35 or with baseline normal ALT levels, TAF remains highly effective in suppressing viral replication, and 96-week of continuous use did not adversely affect patients’ lipids and renal function. Currently, the treatment of CHB is mainly based on NAs drugs. TAF is one of the most commonly used medications in China. Compared with tenofovir disoproxil fumarate, it has the advantages of lower dose as well as higher bone and kidney safety (13); compared with entecavir, it is less prone to drug resistance and low viremia (see Table 8 for more information) (14). Notably, TAF still demonstrated favorable viral response even in CHB patients with normal ALT levels and no liver fibrosis (15). Therefore, this study selectively included CHB patients treated with TAF, and more research is needed to determine whether there are similar results in CHB patients treated with other NAs drugs.

**TABLE 8:** Comparisons of efficacy and side effects of TAF with other NAs drugs.

NAs	Efficacy	Side effects
TMF	Similar with TDF (22, 23), not inferior to TAF (24)	No adverse reaction on blood lipids and renal function (22, 25, 26)
TDF	Effective for CHB (27)	Risk factors for kidney and bone impairment (28, 29)
ETV	Similar with TDF and TAF in treatment-naïve patients with CHB (30)	Drug resistance (31)
TAF	As effective as TDF (32)	Improved renal and bone safety (32)

TAF, tenofovir alafenamide; TMF, tenofovir amibufenamide; TDF, tenofovir disoproxil fumarate; ETV, entecavir, CVR: complete virologic response.

There is a trend toward gradual simplification of the indications for anti-HBV therapy in the world. The first is the revision of therapeutic threshold of ALT. Prior to the launch of the 2019 edition of the Chinese guideline, the threshold of ALT for initiating treatment remained at 2 × ULN. Histologic evaluation for the presence of hepatitis activity is required for patients with ALT lower than 2 × ULN; however, this invasive test is difficult to implement clinically. Subsequent studies found that CHB patients with ALT < 2 × ULN can still benefit from antiviral treatment (16). Thus, China adjusted the threshold to ALT < 1 × ULN after 2019. Of note, hepatitis progression will still occur even in patients with ALT lower than 1 × ULN. For example, liver pathology results of 253 chronic HBV-infected patients with ALT < 1 × ULN suggested significant inflammation (≥ G2) or fibrosis (≥ F2) in 50.2% of patients (17). Moreover, The ALT level still correlates with the severity of hepatitis and long-term prognosis in these patients. Lowering the therapeutic threshold of ALT has now become the main viewpoint of anti-HBV therapy. For example, 30 U/L for men and 19 U/L for women are recommended as the threshold for initiating antiviral therapy, which can effectively improve the CHB treatment rate and help to achieve the goals proposed by the WHO (18). However, whether anti-HBV therapy is still beneficial for patients with ALT < 1 × ULN needs to be further explored. In this study, some of the ALT-normal HBV DNA-positive CHB patients underwent hepatic biopsy. Hepatic inflammation and fibrosis did exist in some of these patients, which was consistent with previous studies. After 96-week follow-up, there was significant difference in viral suppression rate in the ALT-normal group before and after treatment, suggesting that this group of patients can also take TAF to control the level of HBV DNA and improve the prognosis of the disease.

Current national and international guidelines recommended starting antiviral therapy in CHB patients with normal ALT mostly at the age of 30–40 years old (7, 9). The 2019 edition of the Chinese guideline still stated that CHB patients older than 30 years and with a family history of hepatitis B needed to initiate antiviral therapy (11). However, the 2022 version simplified this requirement, and updated it to say that anti-HBV treatment can be started if any of the above conditions are met (19). Given that older patients with CHB are more likely to develop hepatocellular carcinoma or cirrhosis (20), the simplification of the age requirement may help to better control the disease and prolong survival time. In this study, we compared the difference in treatment efficacy before and after TAF treatment between the younger and older age groups using the age of 35 years as the cutoff, and concluded that TAF was effective for CHB patients aged 30–35, which further provided real-world data to support the update of the 2022 edition of the guidelines in China. However, this study also had certain limitations, namely the mismatch in baseline subgroup patient data, which prevented comparison of whether TAF efficacy differs between the low-age group and the high-age group, or the ALT-normal group and the ALT-elevated group. In the future, we will further increase the sample size to refine this result.

In conclusion, in order to better improve the treatment rate of CHB patients, reduce mortality, and achieve the WHO goals, the anti-HBV indications recommended by the current guidelines and expert consensus are gradually expanding and becoming more active. Studies indicated that the “Treat-all” strategy (antiviral treatment for all HBV DNA-positive patients) is cost-effective (21), which can achieve the WHO target of “80% CHB treatment rate by 2030.” This study provided a preliminary reference for the implementation of this strategy, that is, TAF had good efficacy in HBV DNA-positive CHB patients with less impact on lipids and renal function, but whether the “Treat-all” strategy can be realized in the future still needs to be supported by large-sample and long-time follow-up data.

## Data Availability

The original contributions presented in this study are included in this article/supplementary material, further inquiries can be directed to the corresponding authors.
